# Syndrome Differentiation and Treatment Regularity in Traditional Chinese Medicine for Type 2 Diabetes: A Text Mining Analysis

**DOI:** 10.3389/fendo.2021.728032

**Published:** 2021-12-23

**Authors:** Zhili Dou, Ye Xia, Jiawei Zhang, Yizhen Li, Yunan Zhang, Lei Zhao, Zhe Huang, Haonan Sun, Lin Wu, Dongran Han, Yixing Liu

**Affiliations:** ^1^ School of Life and Science, Beijing University of Chinese Medicine, Beijing, China; ^2^ School of Management, Beijing University of Chinese Medicine, Beijing, China

**Keywords:** type 2 diabetes mellitus, traditional Chinese medicine syndromes, acupoint therapy, Chinese herbal medicine, text mining

## Abstract

**Objective:**

The goal of this study was to systematically summarize and categorize the syndrome differentiation, medication rules, and acupoint therapy in the domestic traditional Chinese medicine (TCM) literature on type 2 diabetes mellitus (T2DM), such that guidelines and new insights can be provided for future practitioners and researchers.

**Methods:**

Taking randomized controlled trials (RCTs) on the treatment of T2DM in TCM as the research theme, we searched for full-text literature in three major clinical databases, including CNKI, Wan Fang, and VIP, published between 1990 and 2020. We then conducted frequency statistics, cluster analysis, association rules extraction, and topic modeling based on a corpus of medical academic words extracted from 3,654 research articles.

**Results:**

The TCM syndrome types, subjective symptoms, objective indicators, Chinese herbal medicine, acupuncture points, and TCM prescriptions for T2DM were compiled based on invigorating the kidney and *Qi*, nourishing *Yin*, and strengthening the spleen. Most TCM syndrome differentiation for T2DM was identified as “*Zhongxiao*” (the lesion in the spleen and stomach) and “*Xiaxiao*” (the lesion in the kidney) deficiency syndromes, and most medications and acupoint therapies were focused on the “Spleen Channel” and “Kidney Channel.” However, stagnation of liver *Qi* was mentioned less when compared with other syndromes, which did not have symptomatic medicines.

**Conclusion:**

This study provides an in-depth perspective for the TCM syndrome differentiation, medication rules, and acupoint therapy for T2DM and provides practitioners and researchers with valuable information about the current status and frontier trends of TCM research on T2DM in terms of both diagnosis and treatment.

## Introduction

Type 2 diabetes mellitus (T2DM) is a chronic endocrine and metabolic disorder characterized by either reduced insulin production or insulin resistance resulting in drastically increased blood glucose levels (1). T2DM has been contributing to the burden of mortality and disability worldwide (2). Globally, at least 1 in 11 adults has diabetes mellitus (nearly 90% of them having T2DM pathology), while the T2DM cases in Southeast Asia are projected to reach the 151 million mark by the end of 2045 (3–6). The latest epidemiological survey data indicate that the prevalence of T2DM in mainland China is about 12.8%, according to the American Diabetes Association (7). Since the initiation and progression of diabetes associated with multifaceted comorbid conditions, it has been urgently required to re-optimize the diagnostic and treatment procedures, keeping pace with the advancement of medical sciences and counting in any novel pathological conditions related to diabetes.

For centuries traditional Chinese medicine (TCM) has been proven to be highly effective in treating numerous chronic and critical illnesses, including diabetes, which can be traced back to the theories illustrated in the Inner Canon of Yellow Emperor. Notably, the research has found that the clinical benefits of kai-yu-jiang-zhuo decoction are similar to that of metformin, the most used diabetes medicine (PMID: 25132859). Other studies have shown that Tianqi notoginseng root extract can dramatically reduce the risk of transforming impaired glucose tolerance into chronic T2DM by nearly 32% compared to a placebo treatment (PMID: 24432995). Furthermore, routine treatment with tang-min-ling pills, a combination of ten Chinese herbal medicines, can significantly improve the insulin secretion by pancreatic β cells and reduce the level of fasting blood glucose and glycosylated hemoglobin in patients with diabetes (PMID: 23231379). Since TCM treatments rely greatly on the syndrome differentiation, they, can formulate the most effective personalized herbal medicine regimen. Thus, this strategy has shown potential efficacy in treating T2DM and related disorders in several studies (PMID: 16466178, PMID: 7841750, PMID: 11783182).

In TCM, “*xiaoke*” is the name for diabetes (PMID: 20923535). Classically, diabetes syndromes can be divided into four deficiency syndromes: *Qi* (i.e., life force or vital energy, which belongs to *Yang*) deficiency; *Yin* (i.e., the opposite of *Yang*; e.g., liquid, blood) deficiency and excessive heat syndrome; *Qi* and *Yin* deficiencies and kidney *Yin* deficiency; and two excess syndromes that include blood stasis and phlegm-dampness syndromes (8). In TCM, early-phase diabetes-associated lesions are also classified distinctly as “*shangxiao*” for lung lesions, “*xiaxiao*” for kidney lesions, and “*tripteryg*” for spleen and stomach lesions (PMID: 31704615).

Studies have found that microvascular complications in diabetes influence chronic comorbid complications (PMID: 27230641). Therefore, in practice, TCM clinicians primarily focus on removing blood stasis to improve blood circulation, similar to practices in Western medical therapies (PMID: 23238996). Blood stasis occurs due to *Qi* deficiency, stagnation, and cold retention (PMID: 23163160). Hence, invigorating the spleen and promoting *Qi* using TCM herbs can significantly reduce the risk of diabetic nephropathy (PMID: 28641649).

The treatment for T2DM in TCM includes Chinese herbal medicine (9–12), acupuncture (13, 14), exercise therapy (15–17), and diet (18, 19), with their therapeutic effects supported by previous clinical reports (20). Unlike Western therapies, which mainly focus on blood glucose regulation in patients with diabetes, TCM emphasizes symptom improvement, which can provide long-term benefits and prevent secondary complications (20).

Despite the potential efficacy and importance of TCM applications in the treatment of T2DM, no studies have systematically summarized the research hotspots and fundamental mechanisms underlying this procedure. Randomized controlled trials (RCTs) are the gold standard for evaluating therapeutic effects, and increasingly well-designed RCTs have been conducted worldwide to evaluate the efficacy of TCM-based therapies (21–24). This study intends to systematically review the literature on RCTs for the treatment of T2DM based on TCM and analyze the frequency of medical/academic words utilized in these RCTs by extracting them using text mining with natural language process (NLP) tools.

Natural language processing (NLP) is a field of artificial intelligence that utilizes computers to analyze, understand, and interpret human language. It has received increasing attention in medical research in recent years. For a large variety of unstructured texts such as diagnostic tests, surgical records, test reports, medical orders, progress note, and nursing records, NLP has become an important method and tool for mining clinically useful information in medical texts. For example, some researchers used deep learning Word2vec and TF-IDF to extract text features before, applying association rule algorithm and complex network analysis methods to analyze the compatibility between pairs of drugs and correlations of drugs with symptoms (25–29). In addition, NLP tools have been applied in the field of TCM in recent years. For example, Wei et al. extracted Chinese herbal medicines with high frequencies through data mining of prescriptions in classic TCM literature (30). They applied NLP to extract diagnostic information obtained from tongue images of patients to generate prescriptions by a convolutional neural network (31).

At present, the NLP applications in the field of TCM primarily focus on the unstructured data of medical records, including TCM syndrome, tongue image, pulse condition, prescription drug rule (32–34), whereas there are few researchers who have applied NLP for extracting information in TCM research articles. In TCM research, manual extraction of a large amount of information is still applied to transform unstructured data into structured data, a time-consuming and laborious process, and mistakes can easily be made. In this study, with text mining using NLP tools, the current research status of the focused medical academic words and inter-relationship among them can be discovered, providing novel knowledge and in-depth guidelines for future research on TCM-based treatments for T2DM.

## Methods

### Data Collection

Research articles for the treatment of T2DM in TCM published from 1990 to 2020 were retrieved from the three major Chinese literature databases (Wan Fang, VIP, and CNKI). The following queries were used to search for articles by title, summary, or keywords: (“type 2 diabetic mellitus” or “type 2 diabetes” or “T2DM” or “type II diabetic mellitus” or “ DM II “or “type II diabetes”) and (“Chinese medicine” or “TCM” or “Chinese herbal medicine” or “traditional medicine” or “alternative medicine” or “complementary medicine” or “herbal” or “prescription” or “recipe” or “formula” or “Chinese patent medicine” or “Chinese traditional patent medicine” or “han prescription” or “TCMWM” or “acupuncture” or “needle” or “moxibustion” or “ points” or “syndromes” or “syndrome” or “syndrome elements” or “integrated traditional Chinese and western medicine” or “integrated Chinese and western medicine” or “integrated traditional and western medicine” or “TCMWM” or “TCM-WM” or “integrated TCM WM”).

### Inclusion Criteria

For simplicity and objectivity, only RCTs of T2DM research in TCM were selected to establish the corpus of medical academic words and future analysis in this study.

### Exclusion Criteria

**Figure 1:**
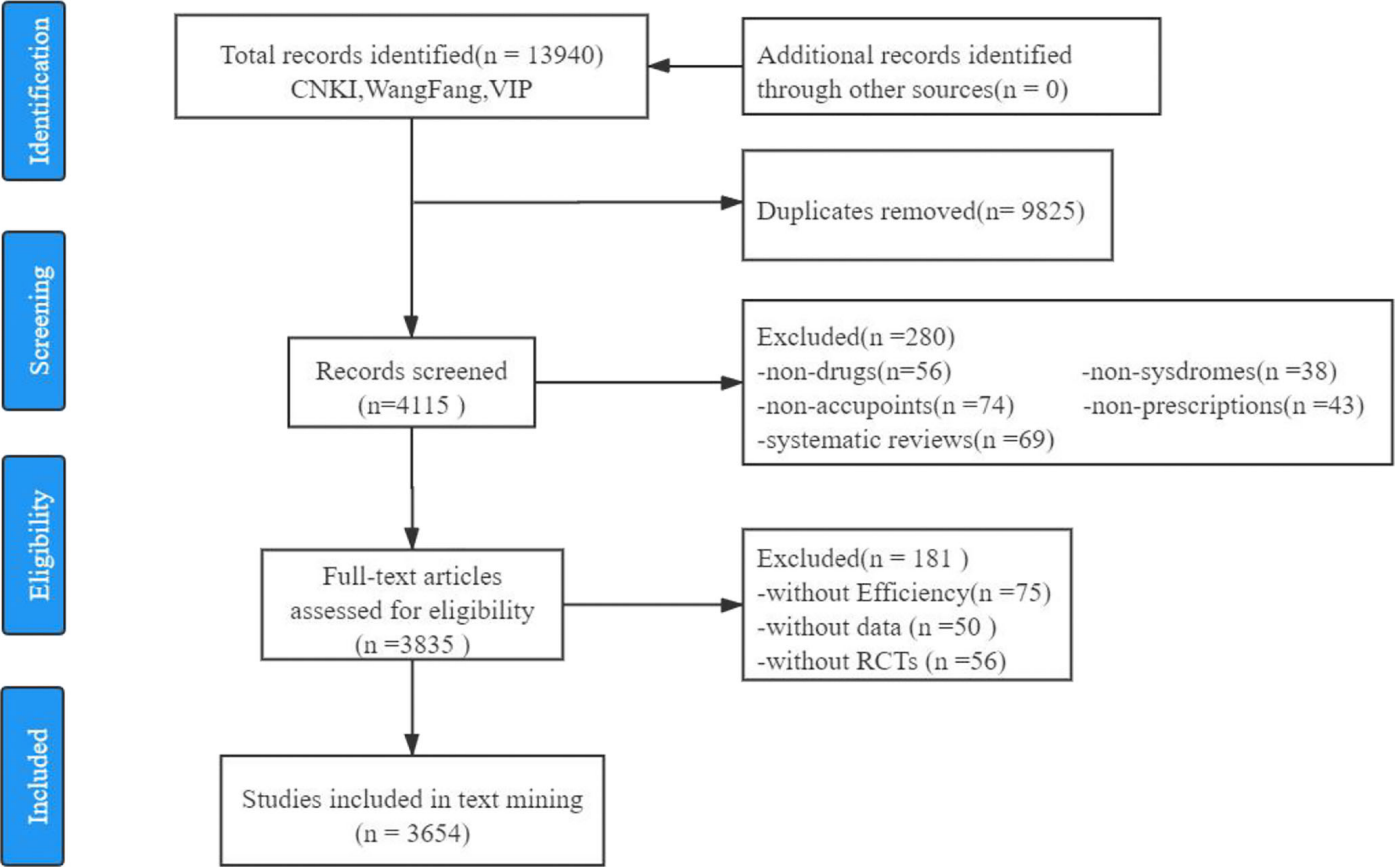
Flowchart for the selection of RCTs.

Articles were excluded if they focused on (1) Type 1 diabetes; (2) only Western medicine treatment; (3) diagnosis; (4) policy; (5) detection; (6) nursing therapy; (7) risk prediction; (8) massage; (9) extraction process of botanical ingredients; (10) patient compliance. Finally, 3,654 published full-text RCTs were selected for the subsequent text mining. The selection process of this study is depicted in **Figure 1**.

### Corpus Establishment

**Figure 2:**
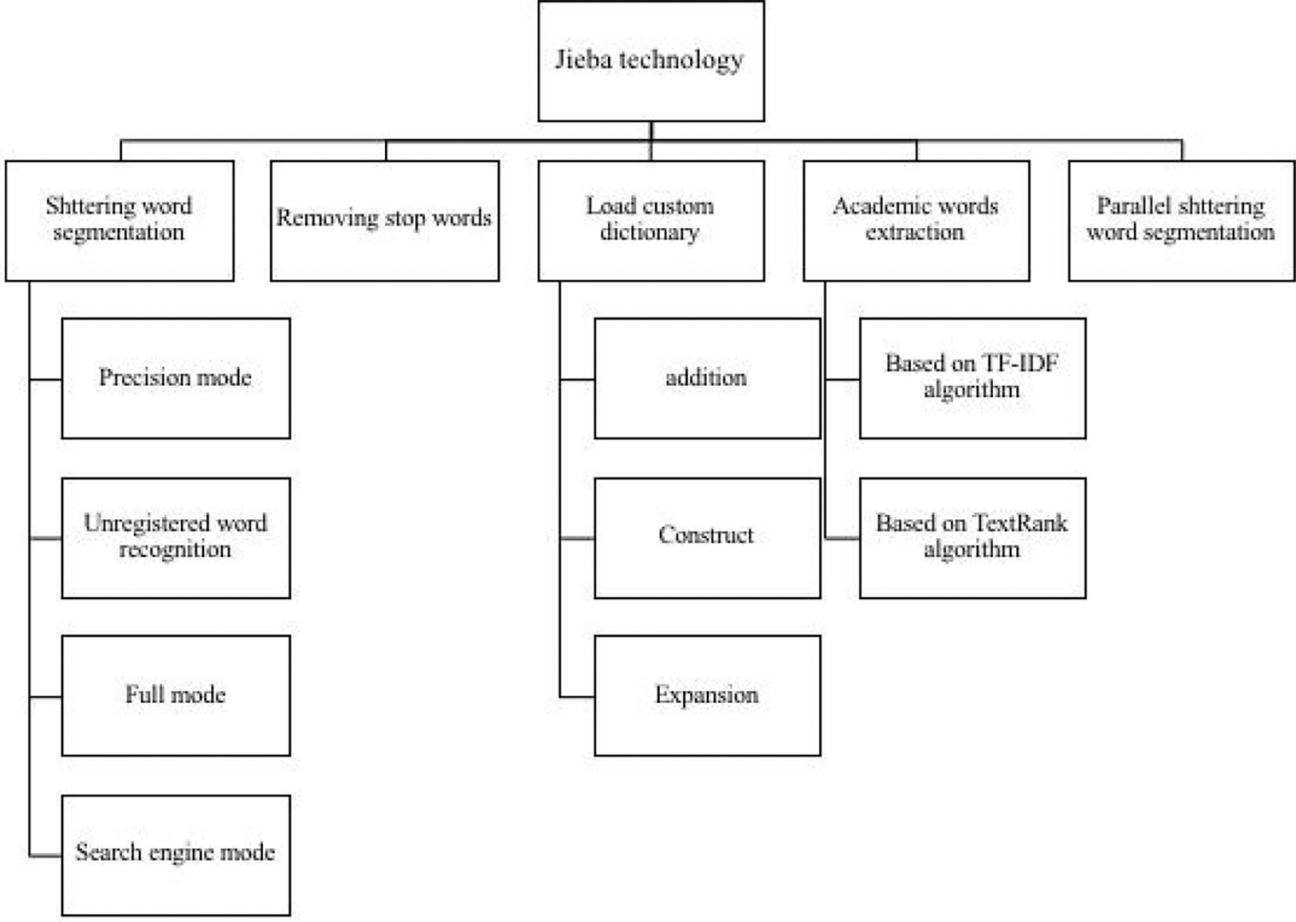
Schematic diagram of stuttering word segmentation. Stuttering word segmentation technology roadmap, from word segmentation, removing stop words, loading custom dictionaries, keyword extraction, part-of-speech tagging to drawing keyword word cloud maps.

Based on the chosen RCTs, we established a corpus of medical academic words with Python 3.7. First, the PDF documents were recognized using the optical character recognition (OCR) technology; then, the focused medical academic words were extracted by segmenting words, removing stop words, and applying a custom dictionary (specific details are shown in **Figure 2**). Furthermore, a total of 41 valid fields were extracted by our automated extraction system, including patient age, group, prescription, herbal medicine, dosage, blind method. The primary rule-based information extraction used in this work is regular expression—a string of texts that allows users to create patterns to match, locate, and manage text—with extracted fields determined by clinical experts of TCM (specific results are shown in **Supplementary 1**, and **Supplementary Figures 1**, **2**). To illustrate the extraction procedure, we took the extraction of TCM prescriptions of T2DM as an example and constructed a binary matrix in which columns are herbal materials, rows represent prescriptions, and each cell has a value of either 0 or 1 (shown in **Table 1**).

**Table 1:** An example of a database constructed from literature.

	Astragalus	Rehammania	Yam	Puerarin	Coptidis rhizoma
**Prescription1**	1	0	1	0	0
**Prescription2**	1	1	0	1	0
**Prescription3**	0	0	1	0	1
**Prescription4**	0	0	1	0	1
**Prescription5**	1	1	0	0	1
**Prescription6**	0	0	1	0	0
**…**					
**Prescription323**	1	0	0	1	0

### Data Analysis

#### Frequency Statistics

We used Python 3.7 for word frequency analysis for each word list categorized as subjective symptoms, TCM syndrome types, TCM prescriptions and herbal medicine, and acupuncture points.

#### Topic Modeling

**Figure 3:**
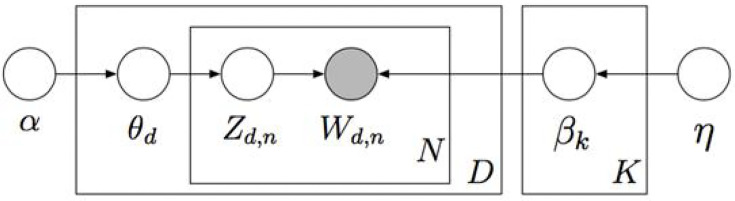
Graphic model for Latent Dirichlet allocation. K, total number of topics; β_k_, topic, a distribution over the vocabulary; D, total number of documents; θ_d_, per-document topic proportions; N, total number of words in a document (in fact, it should be N), Z _d, n_, per-word topic assignment; W_d, n_, observed word; α, 𝔶, Dirichlet parameters.

We used Python 3.7 to conduct topic modeling. The latent Dirichlet allocation (LDA) algorithm was applied for topic modeling (**Figure 3**) to allocate topics to the focused RCTs and words of each RCT to topics based on the Dirichlet distribution. This study used feature weight (TF – IDF = TF × IDF) to evaluate the importance of a word to a text as important evidence for classifying text on the same topic. The TF represents the frequency of a word t*_i_* appearing in the text d*_j_*, expressed by the formula:


$$TF_{i,j}=\frac{{\text{n}}_{i;j}}{\sum_{k}n_{k,j}}$$

The IDF refers to the reverse file frequency, and the calculation formula is:


$$idf_{i}=\log\frac{\left|\text{D}\right|}{\left|\left\{\text{j}:{\text{t}}_{i}\in {\text{d}}_{j}\right\}\right|}$$

where |D| is the total number of files in the corpus, |{j: t*_i_* ∈ d*_j_*}| indicates the number of files containing the word t*_i_*. As indicated by the formula of the feature weight, the more frequently a word appears in one document and the less frequently it appears in another document, the higher the weight and TF − IDF would be.

#### Association Rule Extraction

SPSS Modeler 14.1 was applied to extract association rules for the high-frequency words (i.e., greater than 2%) in subjective symptoms, TCM syndrome types, Chinese herbal medicines, acupuncture points, and TCM prescriptions in each focused RCT. An *a priori* algorithm was applied; the minimum support was set at 10%, minimum confidence at 80%, and the lift at >1.

### Cluster Analysis

In order to cluster the focused RCTs into groups with similar characteristics, SPSS 22.0 was employed to conduct cluster analysis for the occurrence of high-frequency words (i.e., greater than 2%) in subjective symptoms, TCM syndrome types, Chinese herbal medicines, acupuncture points, and TCM prescriptions in each of the focused RCTs. In this study, a hierarchical clustering algorithm with between-groups linkage was applied based on correlation-based distance.

## Results

### The Distribution of Subjective Symptoms

**Figure 4:**
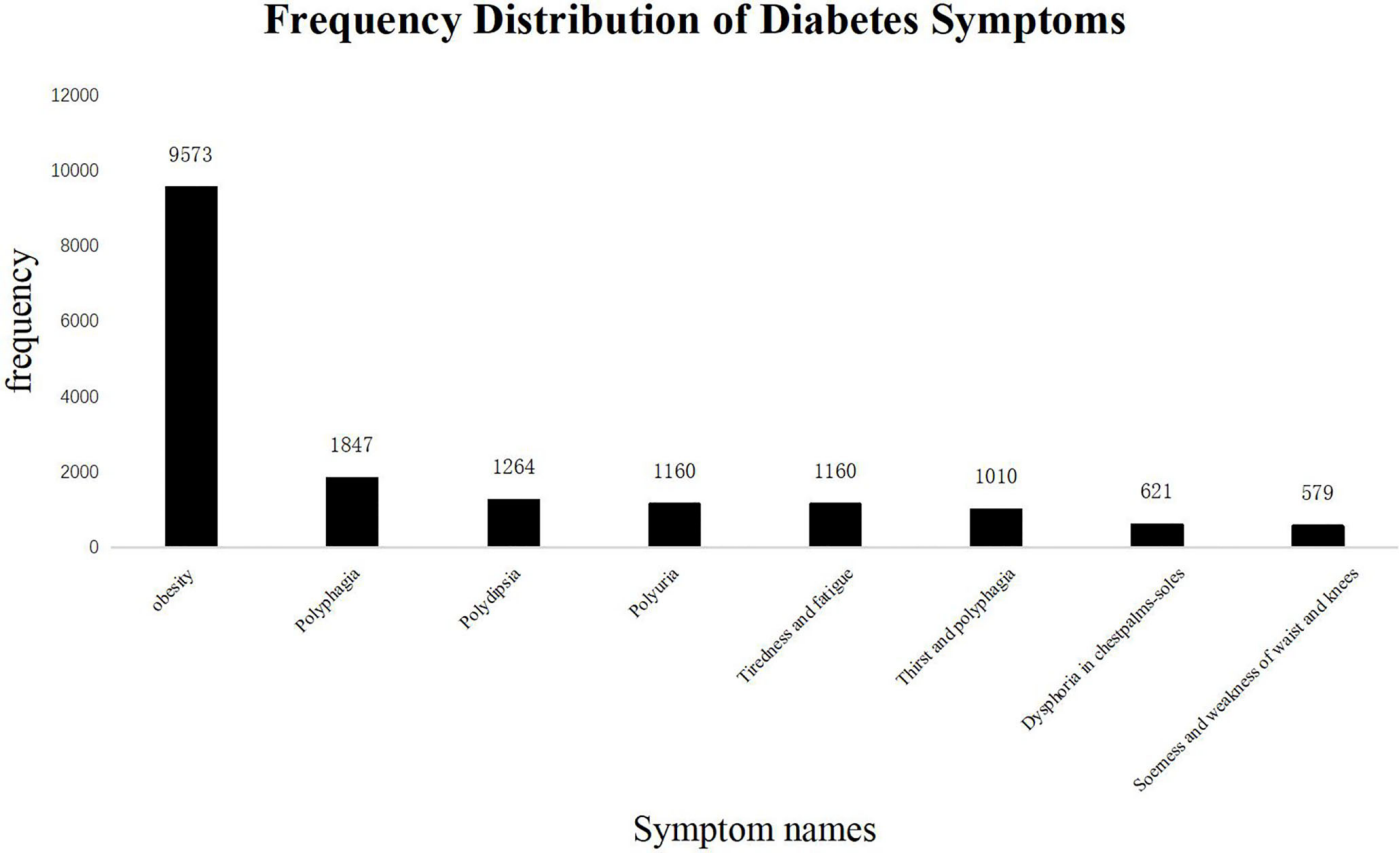
Frequency Distribution of Diabetes Symptoms. The frequency of TCM subjective symptoms in the diabetes literature. The abscissa represents the name of the subjective symptoms, and the ordinate shows the frequency of each symptom. The total number of subjective symptoms of TCM was 27,448, of which more than 2% are as follows: Obesity: 9,573 (34.8%); Polyphagia: 1,847 (6.7%); Polydipsia: 1,264 (4.6%); Polyuria: 1,160 (4.2%); Tiredness and fatigue: 1,160 (4.2%); Thirst and polyphagia: 1,010 (3.6%); Dysphoria in chest, palms, and soles: 621 (2.2%); Soreness and weakness of waist and knees: 579 (2.1%). The most frequent occurrence is “Obesity”, a common symptom of “spleen deficiency and dampness”.

First, we analyzed the distribution of subjective symptoms based on the frequency of the occurrence, applying an arbitrary cutoff threshold of 2% (**Figure 4**). Among them, obesity was the most frequent symptom, followed by polyphagia, polydipsia, polyuria, tiredness and fatigue, thirst and polyphagia, dysphoria in chest, palms, and soles, and soreness and weakness of the waist and knees. From the close observation of frequencies of various symptoms, we found that the nature of the lesion had been focused on deficiencies in multiple energy metabolism systems, and the lesion sites were concentrated in the “spleen” and “kidney.”

### The Distribution of Syndromes

**Figure 5:**
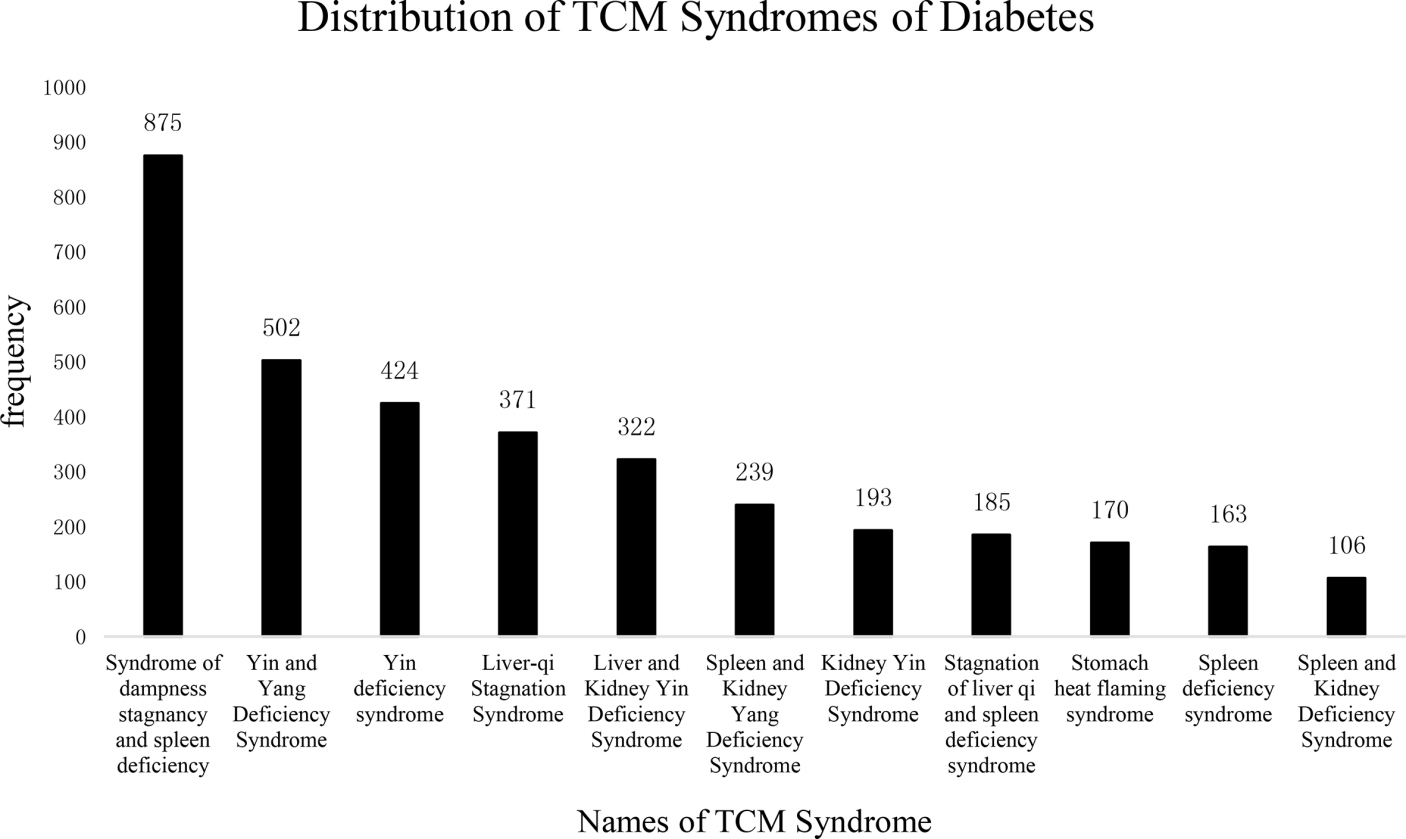
Distribution of TCM Syndromes of Diabetes. The frequency of TCM syndromes in the diabetes literature. The abscissa represents the name of the syndrome; the ordinate shows the frequency of each name. The total number of TCM syndromes was 4,801, among which more than 2% are as follows: Syndrome of dampness stagnancy and spleen deficiency: 875 (18.2%); Yin and Yang deficiency syndrome: 502 (10.4%); Yin deficiency syndrome: 424 (8.8%); Liver-qi stagnation syndrome: 371 (7.7%); Liver and kidney Yin deficiency syndrome: 322 (6.7%); Spleen and kidney Yang deficiency: 239 (4.9%); Kidney Yin deficiency: 193 (4.0%); Stagnation of liver qi and spleen deficiency syndrome: 185 (3.8%); Stomach heat flaming syndrome: 170 (3.5%); Spleen deficiency syndrome: 163 (3.3%); Spleen and Kidney Deficiency Syndrome: 106 (2.2%). From the distribution of syndromes, it can be seen that the “spleen deficiency and dampness syndrome” appears most frequently, which may be related to diabetes the patient’s “polydipsia, polyphagia” is related, the spleen deficiency, and the movement and transformation are unfavorable, which leads to the body dampness, evil, and blood stasis group.

The frequency-based distribution of TCM syndromes is shown in **Figure 5**. Syndromes with the occurrence of higher than 2% were dampness stagnancy and spleen deficiency, *Yin* and *Yang* deficiency, *Yin* deficiency, liver-*Qi* stagnation, liver and kidney *Yin* deficiency, spleen and kidney *Yang* deficiency, kidney *Yin* deficiency, stagnation of liver *Qi* and spleen deficiency, stomach heat flaming syndrome, spleen deficiency syndrome, and spleen and kidney deficiency syndrome. The pathogenic factors mainly were related to “dampness, heat”, which obstructed the movement of *Qi* and blood, leading to “fat”.

### The Distribution of Prescriptions and Herbal Medicine

**Figure 6:**
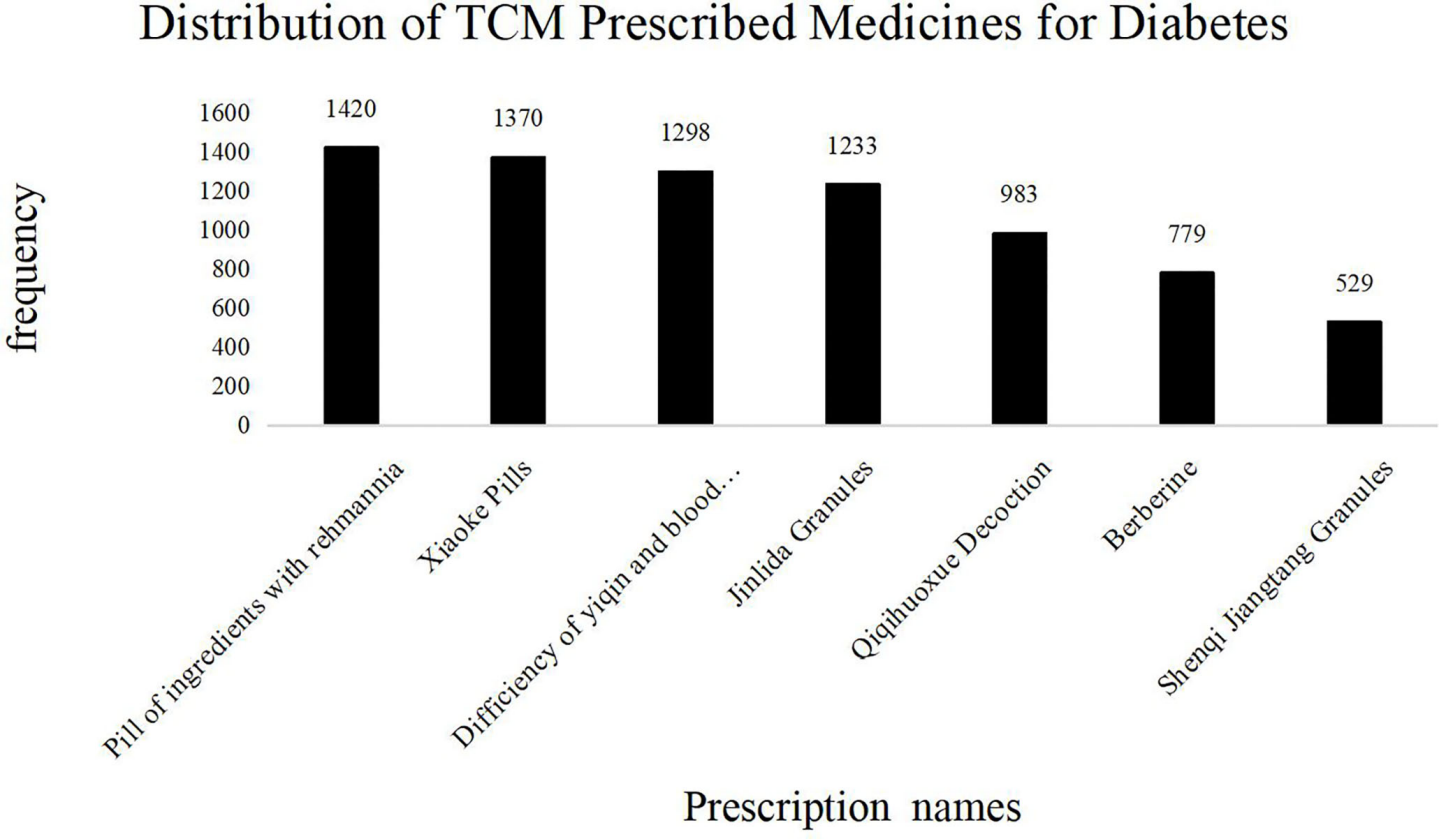
Distribution of TCM Prescribed Medicines for Diabetes. The frequency of TCM prescriptions in the diabetes literature. The abscissa represents the name of the TCM prescription; the ordinate shows the frequency of each prescription name. The total number of TCM prescriptions was 24,748, of which more than 2% are as follows: Pill of ingredients with Rehmannia: 1,420 (5.7%); Xiaoke Pills: 1,370 (5.5%); Deficiency of yiqin and blood unsmooth: 1,298 (5.2%); Jinlida Granules: 1,233 (4.9%); Qiqihuoxue Decoction: 983 (3.9%); Berberine: 779 (3.1%); Shenqi Jiangtang Granules: 529 (2.1%). The most frequently used ones are “pill of ingredients with Rehmannia”. The efficacy of the prescription is consistent with the above symptoms and syndromes.

This research contains 323 prescriptions and 273 herbal materials. The frequency of TCM prescribed medicines for treating diabetic symptoms is shown in **Figure 6**. Medicines prescribed in more than 2% of all literature were (in descending order) *Pill of ingredients with Rehmannia*, *Xiaoke Pills*, *Yiqiyangyinhuoxue Decoction*, *Jinlida Granules*, *Yiqihuoxue Decoction*, *Berberine*, and *Shenqi Jiangtang Granules*. As before, the prescription medicines were mainly composed of Chinese herbal drugs that could invigorate the spleen, replenish *Qi*, and nourish *Yin*.

Most prescriptions (formulas and recipes) comprise several herbal ingredients. The frequency distribution of Chinese herbal medicines based on their application popularity in T2DM treatment is shown in **Table 2**. Astragalus membranaceus (Huang Qi) remains the most frequently used medicinal herb in TCM practice, followed by Rehmannia (Di Huang), Chinese yam, Puerariae (Ge Gen), Coptis rhizome (Huang Lian), Radix Ophiopogonis (Mai Men Dong), Salvia miltiorrhiza (red sage or Dan Shen), Poria cocos (Fi Ling), Radix Trichosanthis (Tian Hua Fen), and Anemarrhena (Zhi Mu).

**Table 2:** Table of classification and frequency of commonly used Chinese medicines for treating diabetes.

ID	Names	Classification	Frequency	Percentage
1	Astragalus	Tonifying and replenishing medicinal	400	5.43%
2	Rehammania	Heat-clearing medicinal	310	4.21%
3	Yam	Tonifying and replenishing	298	4.05%
4	Pueraria	Exterior-releasing medicinal	269	3.65%
5	Coptis	Heat-clearing medicinal	260	3.53%
6	Ophiopogon	Tonifying and replenishing medicinal	250	3.39%
7	Salvia	Blood-activating and stasis-dispelling Medicinal	240	3.26%
8	Poria	Dampness-draining diuretic medicinal	232	3.15%
9	Radix Trichosanthis	Heat-clearing medicinal	224	3.04%
10	Anemarrhenae	Heat-clearing medicinal	222	3.01%

Astragalus (400, 5.43%), Rehmannia (310, 4.21%), Chinese yam (298, 4.05%), Puerariae (269, 3.65%), Coptis rhizome (260, 3.53%), Radix Ophiopogonis (250, 3.39%), Salvia miltiorrhiza (240, 3.26%), Poria cocos (232, 3.15%), Radix Trichosanthis (224, 3.04%), and Anemarrhena (222, 3.01%).

### The Distribution of Acupuncture Points

**Figure 7:**
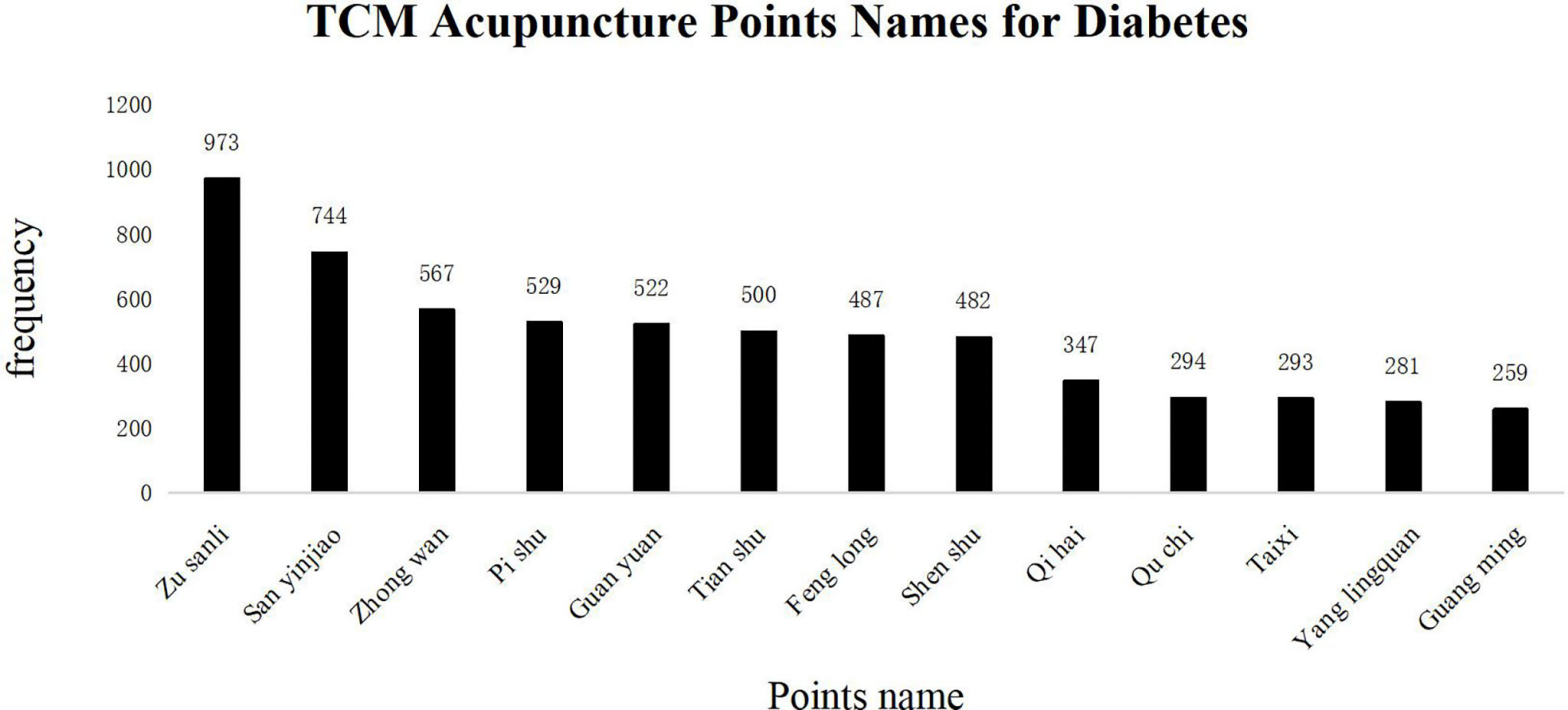
TCM Acupuncture Points Names for Diabetes. The frequency of occurrence of acupuncture points in the diabetes literature. The abscissa represents the name of acupuncture points. The total number of acupuncture points was 12,458, of which more than 2% are the following: ST36: 973 (7.8%), SP36: 744 (5.9%), RN12: 567 (4.5%), BL20: 529 (4.2%), RN4: 522 (4.1%), ST25: 500 (4.0%), ST40: 487 (3.9%), BL23: 482 (3.8%), RN6: 347 (2.7%), LI11: 294 (2.3%), KI3: 293 (2.3%), SP9: 281 (2.2%), GB37: 259 (2.0%). The ordinate shows the frequency of appearance of each acupoint name; ST36 and SP36 are located on the Stomach Meridian of Foot-Yangming and Spleen Meridian of Foot-Taiyin. The abovementioned conforms to the TCM treatment principle.

Commonly used acupuncture points in TCM for T2DM treatments are shown in **Figure 7** in descending order based on their frequency of application in clinical practice, as *Zusanli* (*ST36*), *Sanyinjiao* (*SP36*), *Zhongwan* (*RN12*), *Pishu* (*BL20*), *Guanyuan* (*RN4*), *Tianshu* (*ST25*), *Fenglong* (*ST40*), *Shenshu* (*BL23*), *Qihai* (*RN6*), *Quchi* (*LI11*), *Taixi* (*KI3*), *Yinlingquan* (*SP9*), and *Guangming* (*GB37*). The above acupuncture points are primarily distributed on the Stomach Meridian of Foot-Yangming, Spleen Meridian of Foot-Taiyin, Kidney Meridian of Foot-Shaoyin, and Bladder Meridian of Foot-Taiyang. The principle treatment is directed towards replenishing *Qi*, invigorating the spleen, nourishing Yin, and clearing up the heat.

### Text Topic Extraction

In this study, all included documents were divided into four topic-based categories, and each topic extraction selects the top 10 medical academic keywords with the highest frequency. The results of topic-keywords extraction are shown in **Supplementary Figures 3**, **4** and **Table 1**. We determine the number of topic categories by calculating Perplexity and MPI-score, as shown in our results. When the topic number K was set to 4, there was no intersection between each topic, indicating that 3,654 published RCTs in the literature could be divided into four exclusive topics. Topic 1 was diabetes complicated with cardiovascular and renal dysfunctions, such as urinary protein, glomerulus, proteinuria, diabetic nephropathy, tripterygium wilfordii, urea nitrogen, and chronic nephritis, indicating that these studies were mainly focused on diabetic nephropathy. Topic 2 included non-drug therapy as the theme. In Topic 3, we found diabetic complications related to aging. Moreover, in Topic 4, we observed that obesity and hypertension were the leading symptoms of T2DM.

### Association Rule Analysis

The correlation between diagnosis and treatment information was mined based on the *a priori* algorithm (PMID: 25926855). The minimum support was 10%, the minimum confidence was 80%, and the maximum preceding item was 2. In this study, we used association rule analysis (ARA) to explore the information on the diagnosis and treatment of T2DM. The most common combinations were syndrome of excessive dampness and spleen deficiency with Astragalus membranaceus; obesity and *ST36*; pill of ingredients with Rehmannia extract and *Yin* deficiency; obesity with syndromes of excessive dampness and spleen deficiency. **Tables 3**, **4**, respectively, list the association rules of combining two kinds of diagnosis and three kinds of diagnosis.

**Table 3:** Association rules of two-diagnosis and treatment information.

Consequent	Antecedent	Support (%)	Confidence (%)	Lift (%)
Syndrome of dampness stagnancy and spleen deficiency	Astragalus membranaceus	85.91	96.09	1.03
Spleen deficiency syndrome	Astragalus membranaceus	85.90	90.62	1.02
Qiqihuoxue decoction	Astragalus membranaceus	85.90	90.62	1.16
Obesity	ST36	34.22	100	1.73
Spleen deficiency syndrome	ST36	34.22	92.15	1.04
Stomach heat flaming syndrome	Pill of ingredients with rehmannia	28.92	88.37	1.10
Radix Ophiopogonis	Pill of ingredients with rehmannia	28.86	97.67	1.25
Yin deficiency syndrome	Pill of ingredients with rehmannia	28.86	93.02	1.27
Chinese yam	Pill of ingredients with rehmannia	28.86	100	1.40
Obesity	Pill of ingredients with rehmannia	28.85	86.04	1.49
Poria cocos	Pill of ingredients with rehmannia	28.86	100	3.23
Spleen deficiency syndrome	Syndrome of dampness stagnancy and spleen deficiency	93.28	90.64	1.02
Obesity	Syndrome of dampness stagnancy and spleen deficiency	93.28	82.73	1.03

The results showed that the most common combinations were: syndrome of dampness stagnancy and spleen deficiency with Astragalus membranaceus, obesity and ST36, Pill of ingredients with rehmannia and Yin deficiency, Obesity and syndrome of dampness stagnancy and spleen deficiency.

**Table 4:** Association rules of three-diagnosis and treatment information.

Consequent	Antecedent	Support (%)	Confidence (%)	Lift (%)
Syndrome of dampness stagnancy and spleen deficiency	K13 and Astragalus membranaceus	40.939	93.442	1.001
Spleen deficiency syndrome	Astragalus membranaceus and Chinese yam	60.402	88.888	1.003
Syndrome of dampness stagnancy and spleen deficiency	Puerariae and Astragalus membranaceus	44.966	94.029	1.007
Obesity	ST36 and Astragalus membranaceus	26.845	100	1.732
Obesity	ST36 and syndrome of dampness stagnancy and spleen deficiency	30.872	100	1.733
Syndrome of dampness stagnancy and spleen deficiency	ST36 and Chinese yam	31.543	93.617	1.003
Astragalus membranaceus	Syndrome of dampness stagnancy and spleen deficiency	93.288	88.489	1.030
Astragalus membranaceus	Spleen deficiency syndrome and syndrome of dampness stagnancy and spleen deficiency	84.563	89.682	1.043
Astragalus membranaceus	Puerariae and syndrome of dampness stagnancy and spleen deficiency	44.295	95.454	1.111
Astragalus membranaceus	Radix Ophiopogonis and syndrome of dampness stagnancy and spleen deficiency	44.966	95.522	1.112
Astragalus membranaceus	BL20 and syndrome of dampness stagnancy and spleen deficiency	17.449	100	1.164
Astragalus membranaceus	Spleen and kidney Yang deficiency and syndrome of dampness stagnancy and spleen deficiency	17.449	100	1.164
Astragalus membranaceus	Low spirit and syndrome of dampness stagnancy and spleen deficiency	22.818	100	1.164

### Cluster Analysis

**Figure 8:**
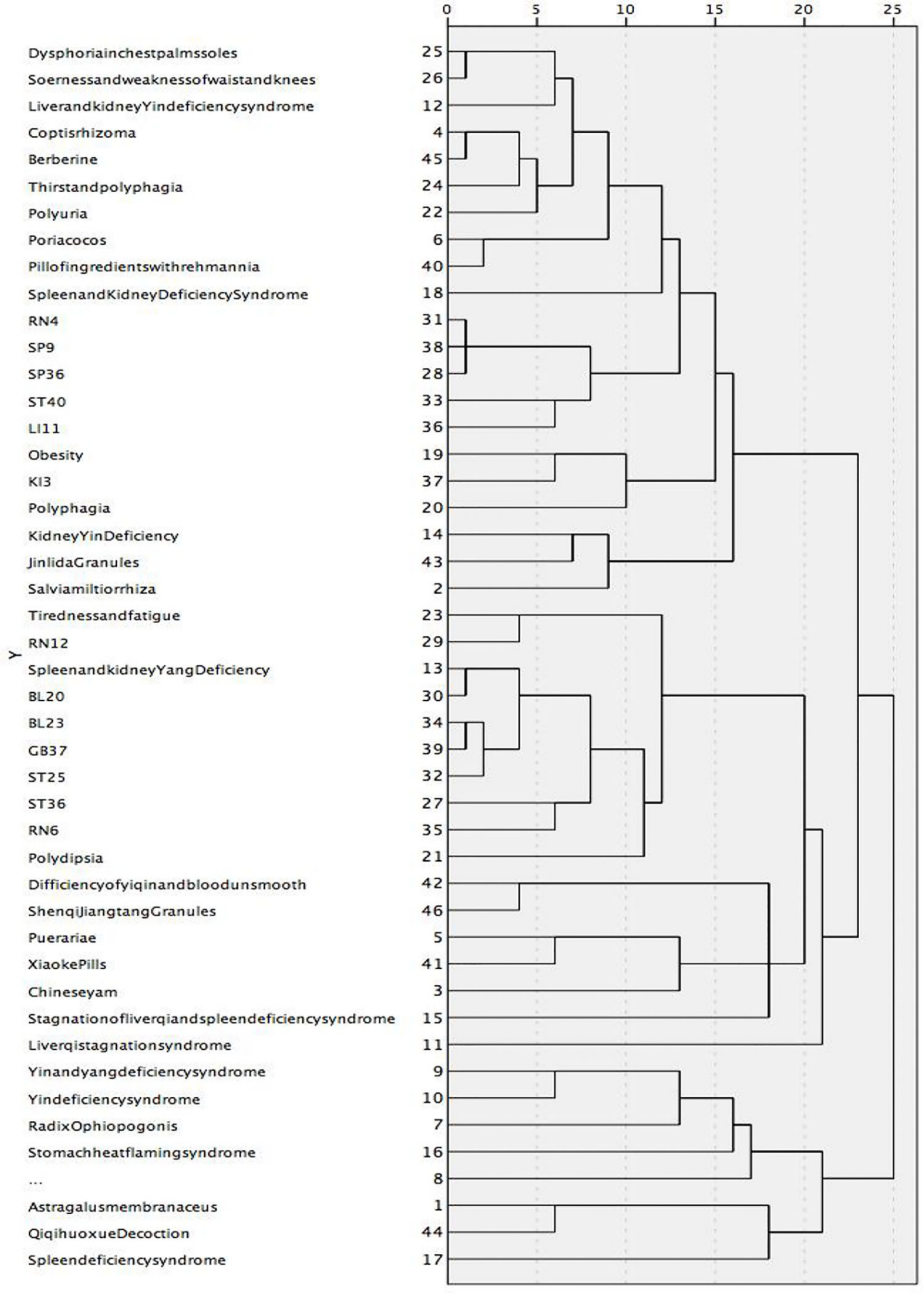
A tree graph using an average join (between groups). Category 1 mainly treated the manifestations of spleen-kidney Yang deficiency syndrome, such as low spirit due to Qi deficiency, polyuria, *BL23* and *GB37*; Category 2 was mainly Stagnation of liver qi and spleen deficiency syndrome; Category 3 was mainly liver and kidney Yin deficiency, such as dysphoria in chest, palms, and soles, thirst and drink, soreness and weakness of waist and knees, thirsty and drinking, *Pill of ingredients with Rehmannia*; Category 4 was mainly spleen deficiency, treatment with Astragalus membranaceus.

The clustering of information in more than 2% of the literature in this study formed four clusters. Cluster-1 mainly treated the manifestations of spleen-kidney *Yang* deficiency syndrome, such as low spirit due to *Qi* deficiency, polyuria, *BL23*, and *GB37*; Cluster-2 primarily focused on the stagnation of liver *Qi* and spleen deficiency syndrome; Cluster-3 mainly addressed the liver and kidney *Yin* deficiency, such as dysphoria in the chest, palms, and soles, thirst and drink, soreness and weakness of waist and knees, thirsty and drinking, pill of ingredients with Rehmannia extract; and Cluster-4 included spleen deficiency and treatment with Astragalus membranaceus. The results are shown in **Figure 8**.

## Discussion

### Subjective Symptoms and Syndrome Differentiation for T2DM in TCM

This study found that “obesity” was the most common symptom for T2DM closely related to “spleen deficiency dampness syndrome,” which is consistent with T2DM pathology in TCM. According to TCM theory, the lack of spleen *Qi* might lead to malfunctioning transport, affecting nutrient transport and water metabolism and leading to obesity. Also, obesity symptoms were related to dampness-heat stasis, and swift digestion resulted from gastric heat.

Our results indicate that the primary phenotypic manifestation of T2DM patients was not categorized exclusively based on the typical “polydipsia, polyphagia, polyuria, and weight loss” symptoms. Interestingly, in addition to obesity, we found the following symptoms in the selected RCTs: (1) tiredness, weakness, shortness of breath, and low spirit due to *Qi* deficiency; (2) thirst, dry throat, dry mouth, and dysphoria with feverish sensation in chest, palms, and soles resulting from kidney *Yin* deficiency; (3) numbness and stasis of the limbs caused by *Qi* deficiency and blood stasis.

According to The Guidelines for the Diagnosis and Treatment of Common Diseases in Internal Medicine of TCM (35) and International Guidelines for the Diagnosis and Treatment of Diabetes in TCM (36), the syndrome differentiation and treatment of T2DM are created according to the deficiency of Yin and Jing (i.e., Qi in concentrated form, which belongs to Yin), and excessive dryness-heat. In addition, there are other categories of TCM syndrome differentiation for T2DM. For example, Internal Medicine of Traditional Chinese Medicine (2^nd^ edition) classifies different syndromes of thirst elimination as “Shang Xiao,” “Zhong Xiao,” and “Xia Xiao.” “Shang Xiao” refers to lung thermo in injury syndrome; “Zhong Xiao” indicates common stomach heat burning syndrome and Qi Yin deficiency syndrome; while “Xia Xiao” is divided into the deficiency syndrome of kidney Yin and the deficiency syndrome of Yin and Yang (37). Furthermore, in Guiding Principles for Clinical Research of Chinese Medicine New Drugs (Trial) (38) and relevant discussion of TCM diagnostics in various versions of TCM internal medicine textbooks (39–41), TCM syndromes of diabetes can be divided wholly into four syndromes: lung heat and fluid injury syndrome, stomach heat and excessive heat syndrome, deficiency of Qi and Yin syndrome, and deficiency of Yin and Yang syndrome. Thus, the pathogenesis of diabetes involves deficiency syndromes and excess syndromes, and deficiency syndromes are mainly manifested in the form of Qi, Yin, and Yang deficiencies.

Like the expansion of symptom corpus, we also expanded the corpus for TCM syndromes of T2DM, which might help TCM practitioners give greater attention to additional subjective symptoms of T2DM patients and interpret their causes from more perspectives. In this study, the top 10 syndromes of TM2D included (1) syndrome of dampness stagnancy and spleen deficiency; (2) *Yin* and *Yang* deficiency syndrome; (3) *Yin* deficiency syndrome; (4) liver *Qi* stagnation syndrome; (5) liver and kidney *Yin* deficiency syndrome; (6) spleen and kidney *Yang* deficiency; (7) kidney *Yin* deficiency; (8) stagnation of liver *Qi* and spleen deficiency syndrome; (9) stomach heat flaming syndrome; and (10) spleen deficiency syndrome. According to the results, the TCM syndrome types of diabetes are complex and dynamic, increasing the difficulty in interpreting them for TCM clinicians. To help clinicians better understand these syndromes, we proposed the following summary: (1) the course of T2DM is considered to be a mixture and alternation of deficiency and excess; (2) the disease lesions are mainly spleen, liver, and kidney; (3) the disease elements are dampness, stasis, and heat. So the primary treatment is for tonifying, nourishing *Yin*, clearing heat, and relieving stasis.

### Prescriptions, Herbal Medicines, and Acupoint Treatment Patterns

T2DM symptoms are mainly manifested as deficiency syndrome, and its fundamental causes are *Yin* deficiency and excessive heat, according to the TCM theory. TCM physicians believe that diabetes pathogenesis is due to the loss of body fluid due to excessive body heat, and patients need treatment to nourish *Yin* and reduce heat. For example, pills of ingredients with Rehmannia extract, which contains Radix Rehmanniae Praeparata, is the appropriate choice to nourish *Yin* and kidney, help produce saliva and quench thirst, clear heat, moisten the lung, and improve both liver and kidney functions (42).

Astragalus was the most frequently prescribed natural drug in our analysis. It has the effect of replenishing *Qi* and raising *Yang* and can generate blood, promote body fluid circulation, cure spleen deficiency, and eliminate thirst. Also, Astragalus and Danshen can be applied together to replenish *Qi*, enhancing the effect of Danshen to promote blood circulation, strengthen the spleen, and reduce dampness and blood stasis. More importantly, modern studies have shown that Astragalus polysaccharide can effectively reduce blood glucose levels, improve insulin sensitivity, inhibit the apoptosis of pancreatic β cells, and play a key role in treating diabetes complications (43). Yam can nourish the *Qi* of the spleen, lung, and kidney and nourish the *Yin* of these organs. Therefore, it serves as an essential medicine for the treatment of T2DM (44). Atractylodes and Poria can invigorate the spleen and reduce dampness, and they are often used to treat T2DM of damp-heat accumulating spleen syndrome (45). Ophiopogon japonicus can nourish the lung and stomach *Yin*; when prescribed together with dogwood, it can nourish liver and kidney *Yin (* 46 *).*

In addition to these drugs prescribed for replenishing *Qi* and invigorating *Jin* (i.e., liquid in the body), the second most prescribed type for T2DM was antipyretic and dehumidifying drugs, whose mechanism has been well studied. Internal fever is believed to be an important patho-mechanism of spleen impairment, and primary attention should be given to eliminating body heat (47). Among antipyretic drugs, Coptis is the most frequently used medicine. Since ancient times, Rhizoma Coptis has been an important anti-thirst drug (48), also known as the “hypoglycemic holy drug.” Modern pharmacological studies have also shown that Rhizoma Coptis can regulate glucose and lipid metabolism, improve insulin resistance, and protect islet β cells (49). Also, Pueraria can clear heat, promote fluid circulation, and quench thirst (50).

Acupuncture and moxibustion—the essential external treatments in TCM—can regulate *Qi*, blood, *Yin*, and *Yang* of the human body by activating acupoints related to respective diseases. Studies have shown that acupuncture at *ST36* can improve insulin sensitivity and the morphology of pancreatic β cells (51). Likewise, activation of *ST40* can improve stomach function. T2DM-associated obesity and hyperlipidemia, related to spleen dysfunction, prevent nutrients from transporting to the whole body, leading to phlegm and dampness accumulation in the waist and abdomen. The ancient TCM book *Song Yu Long* has suggested that acupuncture at *ST40* can help remove phlegm (52).

Moreover, rodent studies have shown that acupuncture at *ST40* can prevent rats from obesity and help them improve fat metabolism and insulin resistance (53). The acupuncture points *BL20* and *BL23* are located on the bladder meridian. Studies have shown that acupuncture at Back-shu acupoint can form a direct neural pathway through spinal ganglia to adjust the functions of internal organs (54). The spleen and stomach belong to “Zhong *xiao*,” interacting with each other, where their chief function is to transport nutrients and water to different parts of the body. The liver and kidney belong to “X*ia xiao*,” whose main function is to channelize gas and regulate mood. It also helps the spleen and stomach transport nutrients, and it improves the normal metabolism of sugar and fat. Animal experiments have shown that acupuncture at *RN12* can increase the expression levels of corticotropin-releasing hormone (CRH) and c-fos protein in the hypothalamus as well as the levels of peripheral insulin and β-endorphin, and it plays a key role in regulating blood sugar (55). *RN4* is the small intestine Mu-front acupoint at Ren meridian and Foot-three Yin meridian intersection point, related to vital energy. *SP36* is the acupoint at foot three Yin meridian intersection points. Chronic T2DM is usually related to deficiency of both *Qi* and *Yin*, along with dryness-heat, which is usually treated by activation of *RN4* and *SP36* acupoints to improve *Qi* and *Yin* for liver, spleen, and kidney jointly.

### Text Topic Extraction Analysis

The LDA extracted the text medical academic words by calculating the TF − IDF value between keywords. When we set K to be 4, we get four separate themes with no intersection.

As shown in our results, diabetes complications are one of the most popular research themes. The T2DM is initially manifested as microalbuminuria and albuminuria and then gradually develops to nephrotic syndrome, and even may lead to renal failure, presenting a progressive aggravation (56). When diabetes induces edema and abnormal urination, diabetic nephropathy (DN) initiates symptoms related to the TCM concept of kidney *Qi* deficiency. Other studies have shown that spleen and kidney deficiency is the key to T2DM, leading to spleen damage and proteinuria (57). Several studies on TCM therapies have shown that patients’ blood glucose indexes can be gradually controlled under comprehensive conditions. At the same time, their blood lipid and insulin resistance will be improved accordingly (58), potentially beneficial to the recovery of patients’ islet cells’ function.

### Evaluation of the Treatment Effects for RCT Articles

Since the efficacy information in the RCT literature is described in various forms, lacking standardization, further breakthroughs are needed to extract them from RCT articles as other core information systematically. Therefore, a random sample of 200 RCTs was selected from the 3,654 chosen RCTs to evaluate their treatment effects to illustrate the overall treatment effects. We found that 94.5% of these articles in the intervention group had an effective rate greater than 70%. Thus, in these research articles, the effective rate in the intervention group was significantly higher than that in the control group, with *p* values less than 0.05, indicating that the diagnosis and treatment information extracted in this study has effective implications for clinical practitioners.

## Conclusion

In this study, NLP technology and data mining methodologies were used to sort out the subject symptoms, classification of TCM syndromes, and treatment methods for T2DM based on unstructured data of RCTs, to make innovative attempts in technical methods and provide a basis for future clinical data research and methodological research. Unlike the theoretical knowledge of syndrome types and treatment methods for T2DM in TCM textbooks or guidelines, we have illustrated the most recent research findings for clinical practitioners. Therefore, syndrome differentiation should be considered comprehensively and flexibly to provide the basis for the diagnosis and treatment for clinicians, rather than only following the theoretical knowledge of textbooks. Furthermore, we have expanded the corpus of common symptoms and syndromes for T2DM, providing researchers and doctors focusing on TCM treatment of T2DM with additional perspectives.

As suggested in this study, the main treatment theories for T2DM in TCM are to invigorate the kidney and promote blood circulation, invigorate *Qi*, and dissipate blood stasis, which is achieved by taking TCM medicine or external treatments (e.g., acupuncture and moxibustion). Different from these TCM treatment methods for T2DM, modern therapies mainly concentrate on proper diet, routine exercise (59), precise medication (60–63), adjustment of lifestyle (64), and other relevant methods (65, 66). Our research findings could also provide insights for researchers and practitioners of Western medicine in the field of T2DM.

Different from another study that analyzed the prescription rules of TCM in the treatment of T2DM based on data mining (67), we found a high frequency of stagnation of liver *Qi* syndrome, and we showed that a pill of ingredients with Rehmannia mainly was used to treat liver dysfunction. Therefore, in clinical treatment, we should pay attention to nourishing *Yin* and invigorating *Jin*, nourishing *Qi* to invigorate the spleen, soothing the liver, relieving depression, clearing away heat, and removing dampness.

In the future, the relationship among symptoms, syndrome, drugs, and efficacy based on the unstructured information extracted in this study can be further explored by using the machine learning algorithm. However, since this study can only be used as a macro-scale study on the distribution of T2DM diagnosis and treatment information, it is necessary to increase the size of the sample of research articles and fine-tune the analytical methods to obtain a more precise pattern for T2DM diagnosis and treatment in TCM.

## Data Availability Statement

The original contributions presented in the study are included in the article/**Supplementary Material**. Further inquiries can be directed to the corresponding authors.

## Author Contributions

YXL, YX, JZ, and DH: Essay writing guidance. YZL, YZ, LZ, ZH, and HS: Data collation. LW: Technical support. All authors contributed to the article and approved the submitted version.

## Funding

This research was supported by the National Key R&D Program of China (No. 2019YFC1710105 and No. 2019YFC1709801).

## Conflict of Interest

The authors declare that the research was conducted in the absence of any commercial or financial relationships that could be construed as a potential conflict of interest.

## Publisher’s Note

All claims expressed in this article are solely those of the authors and do not necessarily represent those of their affiliated organizations, or those of the publisher, the editors and the reviewers. Any product that may be evaluated in this article, or claim that may be made by its manufacturer, is not guaranteed or endorsed by the publisher.
