# Supplementary material for: Syndrome Differentiation and Treatment Regularity in Traditional Chinese Medicine for Type 2 Diabetes: A Text Mining Analysis
Source: Front Endocrinol (Lausanne). 2021 Dec 23;12:728032. doi: 10.3389/fendo.2021.728032 (PMC8733618; doi:10.3389/fendo.2021.728032)
Supplement: Supplementary file 1 [file DataSheet_1.pdf]

Supplementary materials

Supplementary 1

Implementation of file reading and thesaurus establishment code

```
import pandas
#Establishment of corpus, collection of all documents
filePaths=[]
fileContents=[]
#The file directory where the file is located is named root; all subdirectories under root are named dirs; all files un
der root are named files
#Traverse, get all the files needed in the input directory
for root,dirs,files in os.walk('/home/dell/stu/dzl/Meta/txt3654'):
#Get all the files in the root directory, traverse again and append them to the filepaths variable
    for name in files:
        filePath=os.path.join(root,name)
        filePaths.append(filePath)
#Use the codecs.open() method to open each file, use the read() method of the file to read the text in turn, and store
 all the text content in the variable fileContenst in turn，Then the close() method closes the file。
#1. File path, #2. Open method, 3. File encoding
        f=codecs.open(filePath,'r','utf-8')
        fileContent=f.read()
        f.close()
        fileContents.append(fileContent)

#Create data frame corpos, add two variables filePaths and fileContents as an array
corpos=pandas.DataFrame({'filePath':filePaths,'content':fileContents})
corpos
```

Supplementary 2

Fig 1:

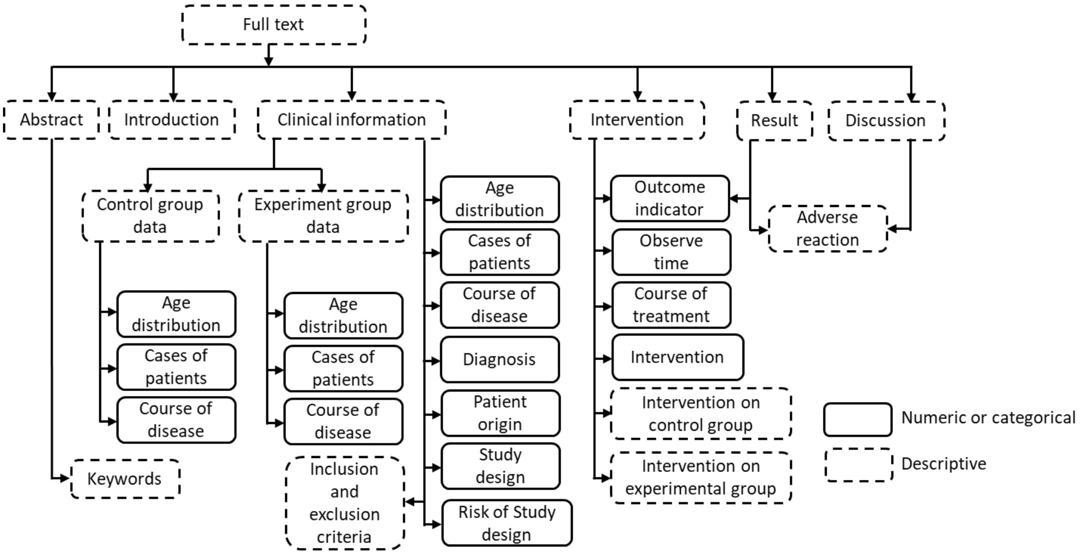

Supplementary 3

Fig 2:

index	title	intermediate	male cases (	female cases	male cases	female case	cases in trea	control grou	The number	The number	Total numbe	Dieasename	chinesesynd	prescription	medicine	effective rate
17	Treatment of	1 Subjects an	not described	not described	not described	not described	not described	not described	not described	not described	not described	Type 2 diabetes		Balsam pear capsules		97.10%
17	Clinical study	1 Materials an	not described	not described	not described	not described	10	10	not described	not described	40	Type 3 diabetes		Balsam pear capsules		96.80%
17	46 cases of ty	1.1 Clinical D	not described	not described	not described	not described	44	44	not described	not described	90	Type 4 diabetes		Balsam pear capsules		95.50%
18	Clinical obser	1.1 Clinical D	22	21	23	20	not described	not described	not described	not described	86	Type 5 diabetes		Balsam pear capsules		95.00%
18	Randomized controlled trial		not described	not described	not described	not described	not described	not described	not described	not described	not described	Type 6 diabetes		Balsam pear capsules		94.80%
18	Effect of Adc	1.1 1 object a	not described	not described	not described	not described	not described	not described	not described	not described	24	Type 7 diabetes		Balsam pear capsules		93.75%
19	Effect of low	1 Clinical dat	10	10	10	10	not described	not described	not described	not described	40	Type 8 diabetes				93.75%
24	Clinical obser	1 clinical data	not described	not described	not described	not described	30	30	not described	not described	60	Type 9 diabetes		Qi Nourishing	Ginseng, Chi	93.62%
24	Treatment of	Clinical data :	22	18	17	13	not described	not described	not described	not described	70	Type 10 diabetes		Qi Nourishing	Ginseng, Chi	93.30%
24	Clinical study	1 Clinical dat	not described	not described	not described	not described	not described	not described	not described	not described	136	Type 11 diabetes		Qi Nourishing	Ginseng, Chi	93.20%
25	30 cases of si	The clinical d	10	20	7	23	not described	not described	not described	not described	30	Type 12 diabetes		Sugar d capsule		92.90%
25	Effects of Ta	1 Data and m	63	37	60	40	not described	not described	not described	not described	200	Type 13 diabetes		Sugar d capsule		92.86%
25	Postoperative	1 Clinical dat	not described	not described	not described	not described	not described	not described	37	29	66	Type 14 diabetes		Sugar d capsule		92.86%
26	Effect of clea	1.1 General D	not described	not described	not described	not described	not described	not described	not described	not described	200	Type 15 diabetes		Compound s	Rhubarb, Co	92.70%
27	Therapeutic	1 Clinical	13	61	10	62	not	not	not	not	146	Type 16		Heat-	Rhubarb,	92.50%
27	Observation c	1.1 General D	14	17	16	15	not described	not described	not described	not described	62	Type 17 diabetes		Heat-clearing,	Rhubarb, Co	92.50%
27	Effect of Qin	1 Data and M	24	22	23	23	not described	not described	not described	not described	92	Type 18 diabetes		Heat-clearing,	Rhubarb, Co	92.00%
31	Effect of Tian	1 Clinical dat	18	13	18	13	not described	not described	not described	not described	63	Type 19 diabetes		Tianmai quench tablet		91.84%
31	Clinical study	1.1 General d	20	10	19	11	not described	not described	not described	not described	60	Type 20 diabetes		Tianmai quench tablet		90.00%
43	Study on imp	Materials and	18	12	18	12	not described	not described	not described	not described	60	Type 21 diabetes		Dan leech glu	Danpi, leeche	90.30%
43	Effect of mox	1 data and me	20	10	19	11	not described	not described	not described	not described	60	Type 22 diabetes		Dan leech glu	Danpi, leeche	89.60%
47	Treatment of	1 clinical data	16	31	9	22	not described	not described	not described	not described	47	Type 23 diab	Qi stagnation	Danji hypogly	rhizoma bletil	89.00%
47	Clinical study	1. Objects an	not described	not described	not described	not described	32	32	not described	not described	32	Type 24 diab	Qi stagnation	Danji hypogly	rhizoma bletil	88.89%
47	Influence of c	1 Subjects an	not described	not described	not described	not described	70	70	not described	not described	140	Type 25 diab	Qi stagnation	Danji hypogly	rhizoma bletil	88.89%
48	Effect of Dan	Subjects and	not described	not described	not described	not described	32	30	not described	not described	32	Type 26 diab	Qi stagnation	Danji hypoglycemic capsule		88.33%
48	Clinical obser	1 Clinical dat	not described	not described	not described	not described	not described	not described	41	45	86	Type 27 diab	Qi stagnation	Danji hypoglycemic capsule		88.33%
49	Intervention c	Materials and	not described	not described	not described	not described	not described	not described	34	26	60	Type 28 diabetes		Danji hypoglycemic capsule		87.50%
49	45 cases of an	1 Clinical dat	24	21	23	21	not described	not described	not described	not described	89	Type 29 diabetes		Danji hypoglycemic capsule		87.27%

Supplementary 4

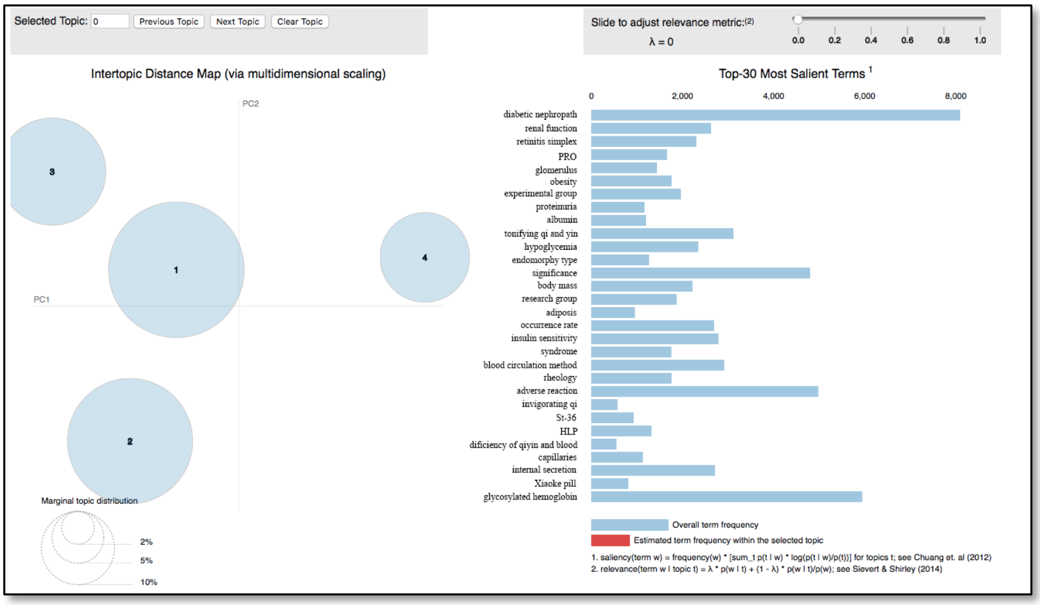

Fig 3: Topic modeling subject word extraction result graph

Topic extraction diagram, the left circle represents the number of topics extracted, the distance between the circle and the circle represents the similarity of the two topics; the bar graph on the right represents the topic words under a certain topic.

Supplementary 5

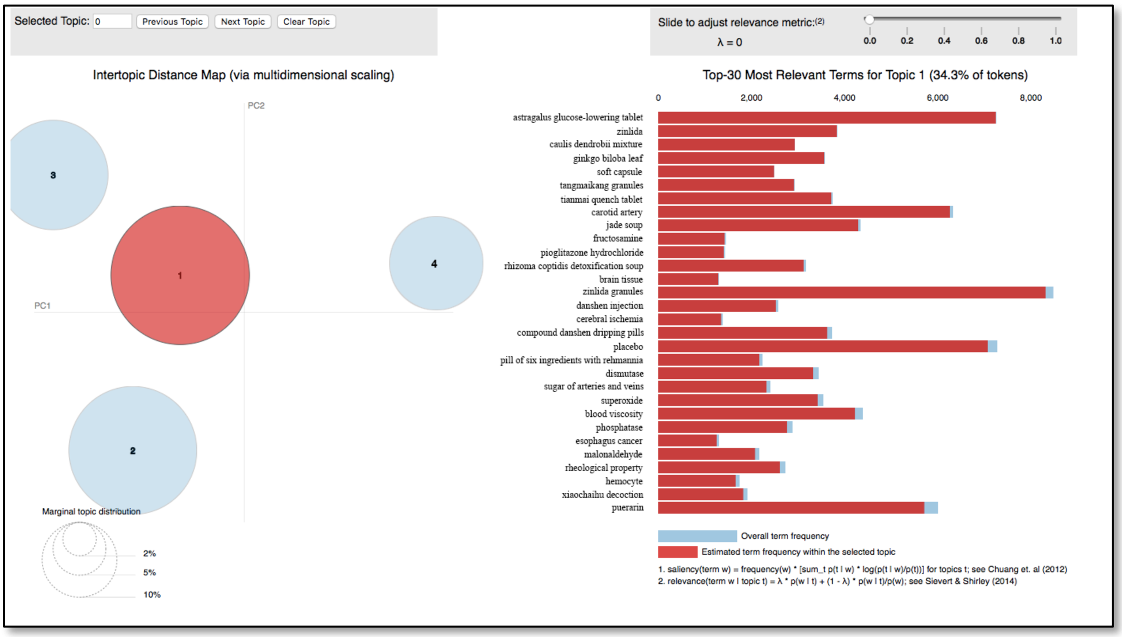

Fig 4: Example image of the subject word extraction results of the LDA model

The red circle on the left represents the specific For a topic, when the mouse is clicked on the topic, the red part of the bar graph on the right is the key word under this topic; Topic 1 literature research disease may be diabetes complicated with cardiovascular disease. Topic 2 takes non-drug therapy as the theme; In topic 3, it can be seen that obesity is also the main symptom of diabetes, and the resulting obesity diabetes is also a clinical research hotspot. Topic 4 focuses on urinary protein, glomerulus, proteinuria, diabetic nephropathy, tripterygium wilfordii, urea nitrogen, and chronic nephritis, indicating that studies on diabetic nephropathy are mainly in this kind of literature.

Supplementary 6

Table 1 Top 10 keywords map for each of the 4 topics

Topic #1:
Diabetic nephropathy, Renal function, Urine protein, Glomerular, supplementing qi and nourishing yin, Proteinuria, albumin, Activating blood circulation to dissipate stasis, Hypertension, Microvascular
Topic #2:
Significant, Metformin, Glucose, Glycated hemoglobin, Insulin sensitivity, Obviously improved, Rheology, Activating blood circulation to dissipate stasis, Collateral, supplementing qi and nourishing yin
Topic #3:
Glycated hemoglobin, Adverse reactions, occurrence rate, Hypoglycemia, The experimental group, Research group, Metformin, Antidiabetic, Average age, Diabetes
Topic #4:
Retinitis simplex, Significance, Obesity, Body fat mass, Endocrine, Syndrome, Endomorphy type, Insulin sensitivity, Hypertension, Adiposis

Represents the top 10 keywords for each topic under 4 themes; All the included documents are divided into 4 topics for keyword topic extraction, and each topic extraction result selects the top 10 keywords whose appearance frequency ranks in the top 10. Topic 1 as gold stilbene hypoglycemic granule, gingko leaf, wheat/day, etc, ginkgo leaf, compound danshen dropping pill and salvia miltiorrhiza injection and carotid artery, brain, cerebral ischemia, blood viscosity together and show theme class 1 literature research disease may be diabetes complicated with cardiovascular disease. Topic 2 takes non-drug therapy as the theme, and the key words include satisfaction, Taijiquan, self-management, nursing staff, Chinese medicine diet therapy, etc, indicating that diet, exercise and self-habit are also commonly used methods for diabetes in such literature. In topic 3, the most concentrated keywords are obesity, obesity, leptin level, percentage of body fat, weight-loss prescription, sex hormones, etc. It can be seen that obesity is also the main symptom of diabetes, and the resulting obesity diabetes is also a clinical research hotspot. Topic 4 focuses on urinary protein, glomerulus, proteinuria, diabetic nephropathy, tripterygium wilfordii, urea nitrogen, and chronic nephritis, From the extraction results, it can be seen that the focus of literature research is diabetic nephropathy and obese diabetes, and the treatment is based on the principle of nourishing qi and yin and promoting blood circulation to remove stasis.
